# RYR 1 Gene Mutation in Motor Neuron Disease: A 10-Year Case Observation

**DOI:** 10.1155/crnm/6050078

**Published:** 2025-10-23

**Authors:** Andreas Posa, Malte Kornhuber

**Affiliations:** ^1^University Clinic and Outpatient Clinic for Radiology and Neuroradiology, Martin Luther University Halle-Wittenberg, Ernst-Grube-Straße 40, 06120 Halle (Saale), Germany; ^2^Neurological Clinic, Helios Hospital Sangerhausen, Am Beinschuh 2a, 06526 Sangerhausen, Germany; ^3^University Clinic and Outpatient Clinic for Neurology, Martin Luther University Halle-Wittenberg, Ernst-Grube-Straße 40, 06120 Halle (Saale), Germany

**Keywords:** electromyography, electrophysiology, motor neuron diseases (MND), myopathy, *RYR1*gene (ryanodine receptor 1)

## Abstract

Motor neuron diseases (MND) are a group of rare, often severe, and life-limiting progressive neurological disorders that primarily affect motor neurons, resulting in muscle weakness and loss of essential muscle functions. Genetic defects play a significant role in MND, contributing to their pathogenesis and progression. The Department of Neurology at Martin Luther University Halle (Germany) followed a male patient with slowly progressive muscle loss, muscle weakness, and muscle pain in the proximal upper arm and shoulder muscles over a period of 10 years and collected clinical, electrophysiological, neuroradiological, laboratory, and genetic data. Clinical neurological and electrophysiological diagnostics clearly indicated MND. A detailed genetic analysis resulted in the first description of an in-frame mutation (heterozygous, c.5691_5693delGGA) in the *RYR1* gene (ryanodine receptor 1), which is unknown in MND or *RYR1*-related neuromuscular disorders. Mutations in *RYR1* are associated with various motor disabilities due to muscle weakness. The specific role of *RYR1* mutations in the genetic pathogenesis of MND has never been described before and is currently unknown. This case is the first of its kind demonstrating a *RYR1* mutation in MND, broadening the spectrum of pathogenetic causes of MND.

## 1. Introduction

Motor neuron diseases (MND) are a group of rare, often severe and life-limiting progressive neurological disorders that primarily affect motor neurons, the nerve cells responsible for controlling voluntary muscle activities such as walking, speaking, breathing, and swallowing [1]. These diseases lead to the malfunction and eventual death of motor neurons, resulting in muscle weakness and loss of essential muscle functions [2]. Currently, several diseases are attributed to MND. The best known is amyotrophic lateral sclerosis (ALS), which is characterized by degeneration of both upper and lower motor neurons, leading to progressive paralysis and respiratory failure [3].

Genetic defects play a significant role in MND, contributing to their pathogenesis and progression. These defects often involve mutations in genes that are crucial for motor neuron function, particularly affecting processes like axonal transport, RNA metabolism, and mitochondrial function [4]. Numerous genes have been identified as contributors to MND.

Genetic testing is becoming increasingly important for diagnosing and managing MNDs, especially with the development of therapies targeting specific genetic subtypes. However, challenges remain due to incomplete penetrance and variants of unknown significance [5]. Mutations in the *RYR1* gene (ryanodine receptor 1) are linked to motor disabilities due to muscle weakness. There is currently no knowledge about the specific role of *RYR1* mutation in the genetic pathogenesis of MND.

## 2. Case Presentation

This article describes the 10 year medical history of a male patient with MND at the Department of Neurology at Martin Luther University Halle, Germany. During this period, repeated neurological, radiological, internal medicine, and oncology examinations were performed. These included a detailed physical examination, electrophysiological measurements (electroneurography, electromyography, and evoked potentials), radiological diagnostics (MRI of the head, shoulder, and complete spine), laboratory chemical diagnostics of blood and cerebrospinal fluid (including neurofilament and antibodies), electrocardiography, and lung function diagnostics. In addition, an extensive genetic analysis was performed.

Symptoms first appeared in 2014 at the age of 55 and fulfilled the clinical diagnostic criteria for MND according to the revised El Escorial criteria. There were clear clinical signs of left-sided upper and lower motor neuron damage (upper motor neuron: left-sided pathologically widened lively muscle reflexes (pectoralis tendon reflex, biceps tendon reflex, triceps tendon reflex, radial periosteal reflex), cloniform expressions; lower motor neuron: clear fasciculations (bilateral; deltoid muscle, biceps brachii muscle, triceps brachii muscle), clear muscle atrophy (bilateral, proximal accentuated, left more than right, strength grade 3-4/5 MRC, deltoid muscle, infraspinatus muscle, biceps brachii muscle, triceps brachii muscle, scapula alata). One year before these symptoms, nonspecific cramps in the upper arm muscles began. The pronounced muscle wasting caused pain in the shoulder and upper arm. There were no other clinical abnormalities (intact: sensitivity, cognition, bulbar area, breathing). Family history was unremarkable. Interestingly, after about a year of rapid symptom progression, the symptoms stabilized and have remained stable since. Despite the symptoms, the patient is still active in his own home and is in full employment.

Detailed electrophysiological diagnostics, repeated annually, consistently confirmed the diagnosis of MND (electromyography [EMG]): proximal and arm-emphasized signs of acute and chronic neurogenic anterior horn damage (high-amplitude action potentials of motor units), pathological spontaneous activity (positive sharp waves and fasciculations), reduced interference pattern: left deltoid muscle, left biceps brachii muscle, right brachioradialis muscle, right interosseous dorsalis muscle, right gastrocnemius muscle, left tibialis anterior muscle, right vastus medialis muscle, left paravertebral L1 muscle; motor evoked potentials (MEP): less activation on the left; somatosensory evoked potentials (SEP): pathological side difference on the left). The muscle-specific blood analysis showed only slightly elevated values for creatine kinase (CK) and myoglobin (MG) (mean values over 10 years: CK: 3.24 (norm: < 3.20 μkat/L); MG: 84 (norm: 28–72 μg/L)).

The only conspicuous finding in the detailed genetic diagnostics was a mutation in the *RYR1* gene (heterozygous change c.5691_5693delGGA) (see Appendix 1 for further findings). Other diagnostic findings (including serum neurofilament light chain protein, NfL) were unremarkable (see Appendix 2).

The initial intake of riluzole (benzothiazole group, sodium channel blocker, and glutamate inhibitor) and its discontinuation after 5 years did not result in any change in the findings. The only available treatment is symptomatic management with physiotherapy and moderate exercise. No other causal treatment is currently available.

## 3. Discussion

Numerous genes have been identified as contributors to MND. These genes often encode proteins involved in axonal transport and RNA metabolism, which are essential for motor neuron function [4, 6, 7]. Mutations in these genes can disrupt these processes, leading to motor neuron degeneration. Mutations in *RYR1* play an important role in neurology by affecting calcium regulation in neurons, which can lead to various neuromuscular disorders. This dysregulation is associated with neuronal phenotypes, as seen in diseases like central core disease, where mutations disrupt voltage-induced calcium release in nerve terminals [8]. Mutations in *RYR1* can manifest in neuronal tissues, indicating a broader impact beyond muscle pathology. This result includes the absence of normal calcium signaling in hypothalamic neurons, which can affect neurological function [8].

*RYR1* mutations are commonly associated with congenital myopathies and can present with neuromuscular symptoms such as muscle weakness and paralysis. These mutations can also lead to atypical periodic paralysis, suggesting a complex interaction between muscle and nerve function [9]. Some *RYR1* mutations are linked to late-onset myopathies, which may include neurological symptoms like axial muscle weakness and spine deformities. These conditions can emerge later in life, expanding the known spectrum of *RYR1*-related disorders [10, 11]. Lawal et al. describe in detail the different subtypes of *RYR1*-related neuromuscular disorders [12].

*RYR1* mutations significantly impact neurological function by disrupting calcium homeostasis, leading to both neuromuscular and neuronal phenotypes. These mutations are associated with a range of disorders, from congenital myopathies to late-onset neuromuscular conditions, highlighting the gene's critical role in both muscle and nerve physiology. This article describes a 10 year follow-up of a male patient with clinical neurological and electrophysiological MND, with a *RYR1* mutation.

The diagnosis of MND in the present case is supported by the clinical and EMG findings, which have been consistent over the years. Clinical findings revealed upper motor neuron damage, with pathologically brisk muscle reflexes and cloniform expressions, as well as lower motor neuron damage, with marked fasciculations and pronounced muscle atrophy of the upper arm and shoulder muscles. The pronounced fasciculations, muscle atrophy, and pathological reflexes, as well as their asymmetric presentation, are atypical for the differential diagnosis of myopathies, particularly *RYR1*-related myopathies. EMG measurements showed neurogenic anterior horn damage with pathological spontaneous activity. Over the years, no evidence of myopathic EMG patterns (small, short-duration polyphasic motor unit potentials with full interference patterns) [13] has been found. The clearly pathological fasciculations, muscle atrophy, and increased reflexes argue against the presence of myopathic inclusion body myositis (IBM), the differentiation of which is sometimes complicated [13]. Unfortunately, the patient repeatedly declined to undergo a muscle biopsy for further diagnostic specificity.  Table 1 presents an explicit comparison of the clinical diagnostic features of previously known *RYR1*-associated myopathies with the clinical findings of this MND case report.

The normal serum NfL values present in this case report do not contradict the MND diagnosis. Elevated values are found for some MND subtypes, such as ALS [16–18], but not for others, such as spinobulbar muscular atrophy (SMBA) [19] or spinal muscular atrophy (SMA) [20]. Due to these differences in serum NfL levels in different MND subtypes, they can be used to differentiate between different MND subtypes [21]. In particular, MND processes with slow progression, as in this case, cause less axonal degradation and thus a lower release of the axonal structural protein NfL into the blood, with normal serum NfL levels despite neurodegenerative pathology [22].

To the best of our knowledge and based on searches in PubMed (scientific database of the U.S. National Library of Medicine—NLM), ClinVar (genomic database of the National Institutes of Health—NIH), and Leiden Open Variation Database (LOVD), the *RYR1* mutation c.5691_5693delGGA described here has not been documented previously. Therefore, its clinical relevance, particularly with regard to *RYR1*-associated myopathies or MND, is not yet known. The classification according to the criteria of the American College of Medical Genetics and Genomics (ACMG) guidelines is provisionally as a “variant of uncertain significance” (VUS, class 3). The heterozygous mutation c.5691_5693delGGA in *RYR1* is an in-frame deletion of three nucleotides within the coding sequence, removing the GGA codon and eliminating a single amino acid from the resulting protein sequence without altering the reading frame. This case report appears to be the first of its kind describing a corresponding *RYR1* mutation in MND. Case reports of gene mutations are essential for better understanding new or rare variants and their clinical context. They contribute to expanded diagnostics by highlighting a possible association between genetic alterations and clinical symptoms, promote scientific exchange, and form the basis for further scientific research.

## 4. Conclusion

This case report describes a slowly progressive adult MND with distinct upper and lower motor neuron signs, with corresponding reproducible electromyographic findings. After 10 years of observation every 6 months and extensive diagnostic workup, no further differential diagnosis could be identified, especially no myopathy. Genetic testing revealed a heterozygous c.5691_5693delGGA *RYR1* mutation. To our knowledge, this is the first description of this variant and the first detection of an *RYR1* mutation in MND that is currently classifiable as a VUS.

## 5. Limitations

The patient reported that no neuromuscular symptoms were known within his biological family. Despite repeated attempts, extended genetic testing of family members using segregation analysis was not feasible, as biological parents and grandparents are deceased, the patient has had no contact with his biological sister for decades, and his two biological children declined both genetic and clinical testing. The unavailability of segregation analysis limits the ability to assess the co-segregation of the identified variant with the phenotype, thereby restricting the strength of evidence for pathogenicity classification according to ACMG guidelines. Consequently, the variant remains classified as VUS.

The patient repeatedly refused a muscle biopsy. This limits the diagnostic workup, as histopathological findings, such as central nuclei, mininuclei, or fiber type mismatch, can provide important clues to a *RYR1*-related neuromuscular disorder. However, the clinical and electrophysiological findings are consistent with MND for years. Two other independent neurological clinics (Neurological Hospital Naumburg [Germany], Neurological Hospital Sangerhausen [Germany]) confirmed the MND diagnosis as second and third opinions based on their own clinical and electrophysiological examinations.

## Figures and Tables

**Table 1:** Comparison of the clinical characterization of *RYR1*-associated myopathies and the MND case report.

	*RYR1*-associated myopathies [12, 14, 15]	MND case report
Onset	Often congenital or in the first years of life	First symptoms at the age of 55

Family history	Often hereditary/positive family history	Unremarkable

Inheritance	Autosomal dominant, X-linked recessive, autosomal recessive, sporadic	Presumably sporadically due to family history

Clinic	Motor development delay	No
	General muscle hypotonia	No
	Emphasis spinal/axial/proximal (especially hip/shoulder girdle muscles)	Yes-proximal emphasis on the shoulder and upper arm muscles
	Muscles weakness	
	Facial/ptosis	No
	Ocular/ophthalmoparesis	No
	Bulbar/dysphagia	No
	Respiratory	No
	Skeletal deformity	
	Thoracic deformity	No
	Scapula alata	Yes
	Scoliosis	No
	Lumbar lordosis	No
	Rigid spine	No
	Contractures	No
	Joint laxity	No

Severity	From unable to walk to barely affected	Independent in own home, fully able to work

Course	Relatively stable/slow progression over longer periods	Stabilization after about 1 year of rapid symptom progression

## Data Availability

The data that support the findings of this study are available from the corresponding author upon reasonable request.

## Appendix A: Unremarkable Genetic Diagnosis

AARS1, ABCA1, ABCD1, ABHD12, ABHD5, ACADM, ACADS, ACADVL, ACTA1, ACTC1, ACTN2, AFG3L2, AGK, AGL, AGRN, A1FM1, A1MP1, ALDH18A1, ALDOA, ALG13, ALG14, ALG2, ALS2, AMPD2, ANG, ANK2, ANKRD1, AN05, AP4B1, AP4E1, AP4M1, AP4S1, APP, APTX, ARHGEF10, ARL61P1, ASAH1, ATL1, ATL3, ATP2A1, ATP7A, ATXN2, B3GALNT2, B4GALNT1, B4GAT1, BAG3, BICD2, B1N1, BSCL2, C0ORF72, C19ORF12, CACNA1A, CACNA1S, CACNB4, CAPN3, CAV3, CAVINI, CCDC78, CCT5, CFL2, CHAT, CHCHD10, CHKB, CHMP2B, CHRNA1, CHRNB1, CHRND, CHRNE, CHRNG, CHST14, CLCN1, CNTN1, COL12A1, COL4A1, COL4A2, COL5A1, COL5A2, COL6A1, COL6A2, COL6A3, COLQ, COQ8A, COX10, COX15, COX6A1, CPT1C, CPT2, CRPPA, CRYAB, CSRP3, CTDP1, CYP27A1, CYP2U1, CYP7B1, DAG1, DAO, DARS2, DCAF8, DCTN1, DDHD1, DDHD2, DES, DGUOK, DHTKD1, DMD, DNA2, DNAJB2, DNAJB6, DNM2, DNMT1, DOK7, DOLK, DPAGT1, DPM1, DPM2, DPM3, DYNC1H1, DYSF, EGR2, ELP1, EMD, EN03, ENTPD1, ERBB4, ERL1N1, ERL1N2, ETFA, ETFB, ETFDH, FA2H, FAM3B, FAM126A, FBLN5, FBX038, FGD4, FHL1, F1G4, FKBP14, FKRP, FKTN, FLNC, FUS, G6PC1, GAA, GAD1, GALC, GAN, GARS1, GBA2, GBE1, GCH1, GDAP1, GFAP, GFPT1, GJB1, GJB3, GJQ, GLB1, GMPPB, GNB4, GNE, GR1D2, GRN, GYS1, HADH, HADHA, HADHB, HARS1, HEXA, HINT1, HK1, HMBF, HNRNPA1, HNRNPDL, HOXD10, HSPB1, HSPB3, HSPB8, HSPD1, HSPG2, IBA57, IGHMBP2, INF2, ISCU, ITGA7, JPH2, KARS1, KBTBD13, KCNA1, KCNE1, KCNE2, KCNH2, KCNJ2, KCNQ1, KIF1A, KIF1B, KIF1C, KIF21A, KIF5A, KLHL40, KLHL41, KLHL9, LICAM, LAMA2, LAMB2, LAMP2, LARGE1, LDB3, LDHA, LITAF, LMNA, LMOD3, LPIN1, LRP4, LRSAM1, MAG, MAPT, MARS1, MARS2, MATR3, MED25, MEGF10, MFN2, MGME1, MPV17, MPZ, MSTN, MTM1, MTMR14, MTMR2, MTO1, MTPAP, MTRFR, MUSK, MYBPC1, MYBPC3, MYF6, MYH14, MYH2, MYH3, MYH6, MYH7, MYL2, MYL3, MYOT, MYOZ2, MYPN, NDRG1, NDUFAF5, NEB, NEFH, NEFL, NEK1, NEXN, NGF, NIPAI, NT5C2, NTRK1, OPA1, OPA3, OPTN, PABPN1, PANK2, PARK7, PDK3, PDYN, PEX12, PFKM, PFN1, PGAM2, PGK1, PGM1, PHKA1, PHKA2, PHKB, PHKG2, PHOX2A, PLA2G6, PLEC, PLEKHG5, PLN, PIP1, PMP22, PNPLA2, PNPLA6, POLG, POLG2, POMGNT1, POMGNT2, POMK, POMT1, POMT2, PON1, PON2, PON3, PPARGC1A, PRKAG2, PRPH, PRPS1, PRX, PSEN1, PSEN2, PYGM, RAB7A, RAPSN, RBCK1, RBM20, REEP1, REEP2, RETREG1, RRM2B, RTN2, RXYLT1, SACS, SBF1, SBF2, SCN2A, SCN4A, SCN5A, SCN9A, SC02, SELENON, SEPTIN9, SETX, SGCA, SGCD, SGCG, SH3TC2, SIGMAR1, SILL, SLC12A6, SLC16A2, SLC1A3, SLC22A5, SLC25A20, SLC25A3, SLC25A4, SLC2A1, SLC33A1, SLC37A4, SLC52A2, SLC52A3, SLC5A7, SMCHD1, SNAP25, SNTA1, SOD1, SOX10, SPART, SPAST, SPEG, SPG2, SPG21, SPG7, SPR, SPTLC1, SPTLC2, SQSTM1, STIM1, SUCLA2, SUCLG1, SURF1, SYNE1, SYNE2, SYT2, TARDBP, TBK1, TCAP, TDP1, TECPR2, TFG, TH, TIA1, TK2, TMEM43, TMPO, TNNC1, TNN12, TNN13, TNNT1, TNNT2, TNNT3, TNP03, TPM1, TPM2, TPM3, TRAPPC2, TREM2, TRIM2, TRIM32, TRPM7, TRPV4, TTN, TTR, TUBA4A, TUBB3, TUBB4A, TWNK, TYMP, UBA1, UBQLN2, VAMP1, VAPB, VCL, VCP, VMA21, VPS37A, VRK1, WASHC5, WNK1, YARS1, YARS2, ZFYVE26, ZFYVE27.

## Appendix B: Unremarkable Diagnostic Tests

Laboratory chemical diagnostics: Neurofilament light chain protein (NfL) (serum: (6 pg/mL, norm: < 45), paraneoplastic and antineuronal antibodies (including AT1A3, CNTN1, Dop. 2, ERC1, GABA-a and b receptors, GluRD2, Homer 3, KCNA2, Neurexin-3, Neurochondrin, Neurofascin, Hu, Ri, ANNA-3, Yo, Tr/DNER, Ma, Ta, GAD 65, Amphiphysin, Aquaporin-4, MOG, Glutamate receptors, NMDA, AMPA, LGI-1, CASPR-2, LON-5, ZIC-4, DPPX, Myelin, CARPVIII, Glycine, mGluR1, mGluR5, Recoverin, Flotillin, ITPR-1, Sez6I2, AP3B2, ThoGT, CV2, PNMA2, Sox, Titin); cerebrospinal fluid diagnostics; electrocardiography; radiological diagnostics (MRI of head, shoulder, and complete spine [no signs of myelopathy]); lung function diagnostics; electroneurographic diagnostics (right median nerve, bilateral ulnar nerve, bilateral tibial nerve, bilateral peroneal nerve, and left sural nerve); internal medicine and oncology assessment.
